# Learners Demographics Classification on MOOCs During the COVID-19: Author Profiling via Deep Learning Based on Semantic and Syntactic Representations

**DOI:** 10.3389/frma.2021.673928

**Published:** 2021-08-02

**Authors:** Tahani Aljohani, Alexandra I. Cristea

**Affiliations:** Computer Science Department, Durham University, Durham, United Kingdom

**Keywords:** learner profiling, MOOC, gender, employment status, CNN, RNN, treeLSTM

## Abstract

Massive Open Online Courses (MOOCs) have become universal learning resources, and the COVID-19 pandemic is rendering these platforms even more necessary. In this paper, we seek to improve Learner Profiling (LP), i.e. estimating the demographic characteristics of learners in MOOC platforms. We have focused on examining models which show promise elsewhere, but were never examined in the LP area (deep learning models) based on effective textual representations. As LP characteristics, we predict here the *employment status* of learners. We compare sequential and parallel ensemble deep learning architectures based on Convolutional Neural Networks and Recurrent Neural Networks, obtaining an average high accuracy of 96.3% for our best method. Next, we predict the *gender* of learners based on syntactic knowledge from the text. We compare different tree-structured Long-Short-Term Memory models (as state-of-the-art candidates) and provide our novel version of a *Bi-directional composition function* for existing architectures. In addition, we evaluate 18 different combinations of word-level encoding and sentence-level encoding functions. Based on these results, we show that our Bi-directional model outperforms all other models and the highest accuracy result among our models is the one based on the combination of FeedForward Neural Network and the Stack-augmented Parser-Interpreter Neural Network (82.60% prediction accuracy). We argue that our prediction models recommended for both demographics characteristics examined in this study can achieve high accuracy. This is additionally also the first time a sound methodological approach toward improving accuracy for learner demographics classification on MOOCs was proposed.

## 1 Introduction

The wave of technological innovations has affected education systems, with one output being the so-called Massive Open Online Courses (MOOCs). They are educational information systems providing a way to democratize knowledge, by usually providing free learning, which successfully attracts significant numbers of users. Owing to this phenomenon, users in MOOCs are very varied in terms of age, gender, employment status, level of education, etc. This diversity makes MOOC environments difficult to navigate; subsequently, this impacts on the learning experience. It becomes important to build personalized recommendations for learners, based on their needs. What is more, many face-to-face courses suddenly stopped during the current pandemic of COVID-19 (WHO, 2020), so the majority of new MOOC users this year are those who are trying to find replacements for their suspended classes (Shah, 2020)—making MOOCs an optimal alternative, as they offer classes from the world’s top institutions (Robson, 2020). According to a recent statistical report (Shah, 2020), enrollments at Coursera, a United States MOOC provider, have increased by 640% just between mid-March to mid-April 2020 (10.3 million in 30 days), compared with the same interval in 2019. Another example in the United Kingdom is FutureLearn, which has now 13.5 million users (Fox, 2020). Currently, MOOCs have proven to be an effective solution for crisis management in the education systems. However, this is shaping the future of MOOCs and emphasizing the importance of improving these platforms to be prepared as an emerging system of education during the pandemic (now), and beyond. In order to improve this critical avenue of no-barriers education, it is important to build information systems providing personalized recommendations systems for learners based on their personal needs. Demographic characteristics are critical inputs into personalized systems. Although MOOCs have open surveys for learners to specify their demographic data during registration, the actual percentage of learners who fill them in is extremely low. Thus, adaptive educational services for MOOCs based demographic data would only be applicable to very few—unless an automatic mode to identify user demographics is explored. In our work, we investigate learners’ posts from a different angle than other works in MOOCs. Our research target is the heterogeneousness of MOOC environments, in terms of their learner demographics based on *employment status* and *gender*. Many studies were concerned with students’ classification in MOOCs, but almost all of these studies used pre-course open responses to identify learners’ characteristics, to be utilized later for different research aims. However, there could be a bias in using such pre-course surveys, as well as sparse data, in the case of non-response. Instead, we aim to predict these learner demographics automatically. Our main research questions are: *1-How can deep learning methods be designed to predict the demographic characteristics of learners in a MOOC, based on the comments they exchange in the discussion forum? This has been done by ultising Ensemble learning of CNN and RNN for employment profiling, and Recursive NN for gender profiling, with both models fed by textual features extracted from comments. 2-What are the most important demographics in MOOCs that are needed for almost all MOOC researches? We discussed in our investigation about the importance of employment and gender as demographics variables for MOOC researches and studies. Most people who take part in MOOCs, particularly, are seeking knowledge or expertise that can enable them to earn a career, and this could range from basic language learning to specialised technical IT skills. MOOCs are great for staying current with industry and market developments, especially as the world is concentrated more on business analytics, artificial intelligence, and other technology-oriented topics and concepts. Furthermore, the pandemic has resulted in mass unemployment, which will make MOOCs a great source for building a competitive workforce. Also employment status is a considerable factor for completing courses in MOOCs (Morris et al. 2015), because of the fact that the majority of those who complete their courses are non-working learners (including unemployed, job-seekers, pensioners, young learners, etc.). The obvious explanation could be that some of them join MOOCs as they (still) need to gain or improve their skills. Especially the ones seeking employment may be interested in obtaining certificates to support their Curriculum Vitae. In addition, much research in MOOCs relies on gender information as a parameter or variable. For example, course content could be personalised based on gender differences, since these differences have been already proven to be one of factors for completing a course in MOOCs, but there are differences which depend on the course (Morris et al. 2015).*

## 2 Related Works

### 2.1 Extracting Demographics Data

Analyzing the writing style of an author, to identify, from a given set of authors, which author has written a particular text, has started since the birth of writing. The sociolinguistics community has applied it to literary and historic texts, like Shakespeare’s works, to determine the linguistic patterns, and was restricted for a long time to this domain. However, the advanced innovations in technology and their extensive influence on growth of online social platforms created more texts than other avenues on the internet. Users can write on these platforms without having to provide their profiles, so users in most cases are unknown. The enormity of user-generated data brings with it many problems of different scales, like plagiarism, or even more serious ones, like online crime. Thus, a new research direction appeared, namely Author Profiling (AP) (Argamon et al., 2009). AP has a wide applicability to many problems from different fields, such as forensics or marketing (Rangel et al., 2013). AP is usually defined as a text classification task within the area of Natural Language Processing (NLP). Due to the incredible amount of users on online platforms, researchers classify these users into groups, based on their similar demographic characteristics, extracted from common text features, learned *via* machine learning algorithms. Here, we apply AP (or LP, i.e. Learner Profiling) on a MOOC, by using our large-scale data collection, including courses delivered by the University of Warwick *via* the FutureLearn platform, to predict the employment status of learners, as well as their gender, so that pre-course questionnaires with a high cognitive overhead, to extract LP, could become redundant.

### 2.2 MOOCs Personalization

It is important to mention here that, according to (Kellogg et al., 2014) and (Reich et al., 2015), the most common type of learners who are attracted to MOOC platforms are those who are aiming to enhance and improve their work-or professional skills. This fits one of the MOOC objectives, to offer to a democratized education for all, especially for those learners who are economically unable to afford a high-quality education—which expands their career chances. In terms of MOOC personalization potential, gender is promising, as researchers have found (Bayeck, 2016) and different behavior whilst learning in MOOCs (Almatrafi and Johri, 2018). For example that males and females are distinct in terms of the type of courses they take. Such differences are inherited from traditional education, where males were shown to prefer Science, Technology, Engineering, and Math (STEM) courses to a higher degree than females (Bayeck, 2016). In addition, the *gender* collaboration pattern in MOOCs is unlike their behavior in traditional learning (Almatrafi and Johri, 2018). Males and females are different in their respective length of active periods and certification rate (Almatrafi and Johri, 2018). It has been found that females were more active in courses in general and obtained higher certification rates than males in non-science courses (Pradubwate et al., 2020). Furthermore, *employment status* is a considerable factor for completing courses in MOOCs Morris et al. (2015), as the majority of those who complete their courses have been found to be non-working Morris et al. (2015). The obvious explanation could be that they still need to improve their skills and they may be getting certificates to support their Curriculum Vitae. In addition, they may have more free time. We have excluded the retired from the not working group in this study because they are obviously are the most learners who have free time. Even they are not working, but they did not consider in this study under the non-worker’s category, so this not affecting the prediction model.

Much research in MOOCs relies on gender information as a parameter or variable. For example, course content could be personalized based on gender, since these differences have been already shown to influence course completion in MOOCs; however, differences may vary based on the course Morris et al. (2015). Hence, auto profiling of learners’ gender, which is this study do, providing important information that could be used to strengthen such study at course level in MOOCs; without do a survey to get the learners gender.

### 2.3 Stylometry Features

It is important to examine the features of the data that are able to distinguish an author’s traits, based on his/her writing style, and then create a set of features that could be used to improve a classifier’s efficiency. The features that can be used for analyzing an author’s writing style are called **Stylometry Features**. This terminology covers a wide spectrum of features, such as lexical features, syntactic features, structural features, readability features, etc. In general, these features are normally characterized by researchers into five levels: lexical, structural, semantic, syntactic, and domain (or content)-specific (Neal et al., 2017). Lexical features are the simplest form of data feature representation, which deal with word and character levels. Both levels can capture differences in style and contextual information. Semantic features are able to capture meaning of words, phrases, and sentences (Neal et al., 2017), and they have an important role in recognizing traits of author (Franco-salvador et al., 2013). Syntactic features, such as word morphology, that dealing with the internal structure of words within a sentence are also an important factor in analyzing an author’s style of writing, and they have been used in some AP studies. Research showed that semantic features and morphological (syntactic) features are excellent factors to distinguish between classes of an author (Argamon et al., 2009). Structural features represent the organization of a document (Neal et al., 2017). These features can be used in long documents, like emails, which sometimes contain signatures, but it is uncommon to find them in short sentences, like tweets. Domain/content specific features refer to features that only applicable to a specific domain, for example mentions in Twitter (Neal et al., 2017).

In our study, we firstly focus on commonly used type of these stylometry features, the semantic representation; additionally we also consider the more uncommon type, the syntactic type. Importantly, we aim at discovering and fine-tuning these representations *via* deep learning models, as such models have not been explored enough with AP, in general (Antkiewicz et al., 2017), and with LP in particular. In addition, such features need to be converted/represented effectively before they are processed automatically by machines. In this step, data will be converted to vectors, then fed to a machine learning algorithm, to perform the classification task (Reddy et al., 2018). Once the data is transformed into vector representations, it will be fed to a classifier, as input for its training. Many NLP studies taking advantage of deep learning methods, which require no handcrafted features (feature engineering) To learn these different types of textual representations.

## 3 Dataset

We collected comments from courses available in FutureLearn^1^, a MOOC platform founded in 2012 (Aljohani and Cristea, 2021), with more than eight million learners just before 2020, that provides courses structured around weekly basis. It is a European online learning information system that provides free learning, which is similar to the American platform, Coursera. FutureLearn is a collaboration between many British universities, the British Library, and the BBC, started in 2012. Since then, thousands of courses have been delivered by many international institutions, businesses, and NGOs, creating an even greater expansion of the platform. We collected comments from courses delivered by the University of Warwick (2013–2017), from different domains, such as social sciences, literature, and computer science, as follows: The Mind is Flat, Babies in Mind, Supply chains, Big Data, Leadership for Healthcare, Literature and Mental Health, and Shakespeare and His World. Theses courses were delivered repeatedly in consecutive years (called Runs), resulting in a total of 27 runs (Aljohani and Cristea, 2019). They have weekly learning units, which cover articles, videos, quizzes and other pedagogical resources. Each weekly learning unit consists of several steps, which can be an article, discussion, video or a quiz. The website also allows learners to comment on any given step. In each weekly learning unit and on any given step, students can “comment”, “reply” and “like” other comments from other users enrolled within the course. In addition, when a learner creates an account at FutureLearn, they have the option to complete a survey about their demographic characteristics, or could complete it later, in case they had skipped this step during the registration. The aim of this survey is to extract information about learners, like gender, age group, education level, and employments status. In addition, the system generates logs, which record all learner activities, like steps, visit times, steps completed, or comments, that are correlated with the unique IDs of learners. However, for our research experiments, we only fetch comments of these learner IDs associated with their labels. This data is currently used for the Author Profiling in our researches. The classes in our data vary in size, because when we have collected comments, we only fetched comments of authors who had already filled in the questionnaires and for whom the author characteristics were known—so we have a labeled dataset. This meant however that the dataset was reduced significantly from the original size. So, we start our experiments with the largest classes in term of the size—employment status and gender.

### 3.1 Employment and Gender Profile

Based on the Employment profile/class, we gathered the user’s ID and comments from these 27 runs of seven courses, totalizing 381,298 comments from 9,538 users. As said, this data is labeled by the learners themselves, based on open-survey at the beginning of each course. There were several types of work statuses recorded in FutureLearn (eight categories): Retired, Working Part-Time, Working Full-Time, Not Working, Self-Employed, Looking for Work, Unemployed, and Full-Time Student, as defined by the FutureLearn platform for the options available. For simplification and compatibility with other studies, we further grouped these into more general types for the professional profile, as follows: retired, working, and not working. This was done due to aiming at a higher accuracy in prediction, as well as due to the fact that some of the original fine-grained FutureLearn statuses were hard to differentiate and slightly ambiguous such as looking for work vs. unemployed, etc. Using a dataset of comments, we wish to learn an embedding function that estimates whether the text of a given comment originates from an employed, retired or unemployed user. For experimenting with another learner characteristic, we also have collected the gender of learners. We obtained about 322,310 samples (265,582 for Females and 56,728 for Males). We used these profiles as targets for our predictive models.

### 3.2 Dealing With Bias

As our data was labeled based on pre-course questionnaires, filled-in by the learners themselves, this represents traditionally higher human accuracy of the labeling. Also, this way of obtaining labeling data is used widely in ML researches. We started by the relatively basic separation of the data into training, testing and validation set. To further avoid any bias (e.g., by learning about the user instead of the type of user) in our training we ensured that no comment written by the same user was included in both training and validation set. This warrants independent samples in both training, testing and validation, to evaluate the model generalisability and achieve unbiased results.

**FIGURE 1:**
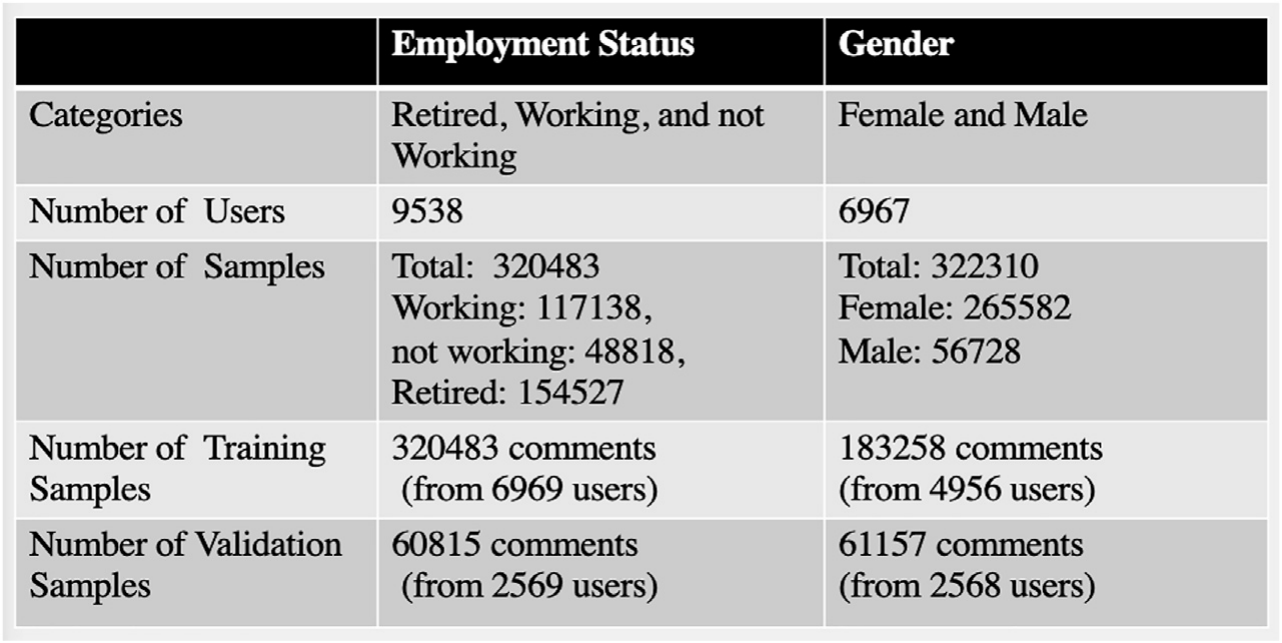
Data Distribution (gender and employment status).

We collected the comments from only one run from each course for the validation dataset. This is because in each run there is a new group of learners. Also, this provided us with enough samples for the validation set. We used data from remaining runs for the training and testing. For the professional profile, we had in total 60,815 comments (from 2,569 users) used to validate the model and 320,483 comments (from 6,969 users) used for training and testing. For the gender profile, we had in total 61,157 comments (from 2,568 users) for validation and 183,258 comments (from 4,956 users) for training and testing, Figure 1. Moreover, to obtain the same class proportion on the training and testing set, after balancing the data as will be explain in the next section, we used stratified sampling, which separates the observations into homogeneous groups (by label) before sampling.

### 3.3 Text Augmentation

The original training set was unbalanced; for the work status class: retirees were 154,527 samples, workers were 117,138 samples, and non-workers were 48,818 samples, while for the gender class: females were 149,904 samples, and males were 33,354 samples. So, we used a technique called text augmentation, for oversampling the training set. This also helps to further reduce the bias of the model in terms of removing the tendency to predict in the majority category. Here, we have used two methods. One is the popular duplication technique of random oversampling (Chawla et al., 2002). However, this did not support the model performance, as will be explained later in this paper. The second, more refined technique applied, was that of paraphrasing (Ganitkevitch et al., 2013). We paraphrased sentences from the lesser size categories to train the model. To do so, we tokenized the large comments, using “.” for tokenization from those minority groups and paraphrased these tokenized sentences, until we achieved the same number of instances in the training set. In other words, we replace words by their synonyms and expressions by their paraphrases to generate new comments. In this last case, we used the paraphrase database PPDB (Ganitkevitch et al., 2013), which has over a billion paraphrase-pairs in total, covering several languages. The idea behind this database is that if two strings S1 and S2, written in a language *A*, have the same translation *f* in another language *B*, then the pair <S1,S2> has the same meaning. As such, <S1,S2> can be extracted as a pair of paraphrases. After we applied this method to balance the training dataset for the work status class, we obtained a training set with (463,581 samples). Then we separated it by the stratified sampling to get 20% of the data for testing (92,716 samples) and the remaining (370,865 samples) for the training.

## 4 Text Pre-Processing

### 4.1 General Text Normalization Steps

**FIGURE 2:**
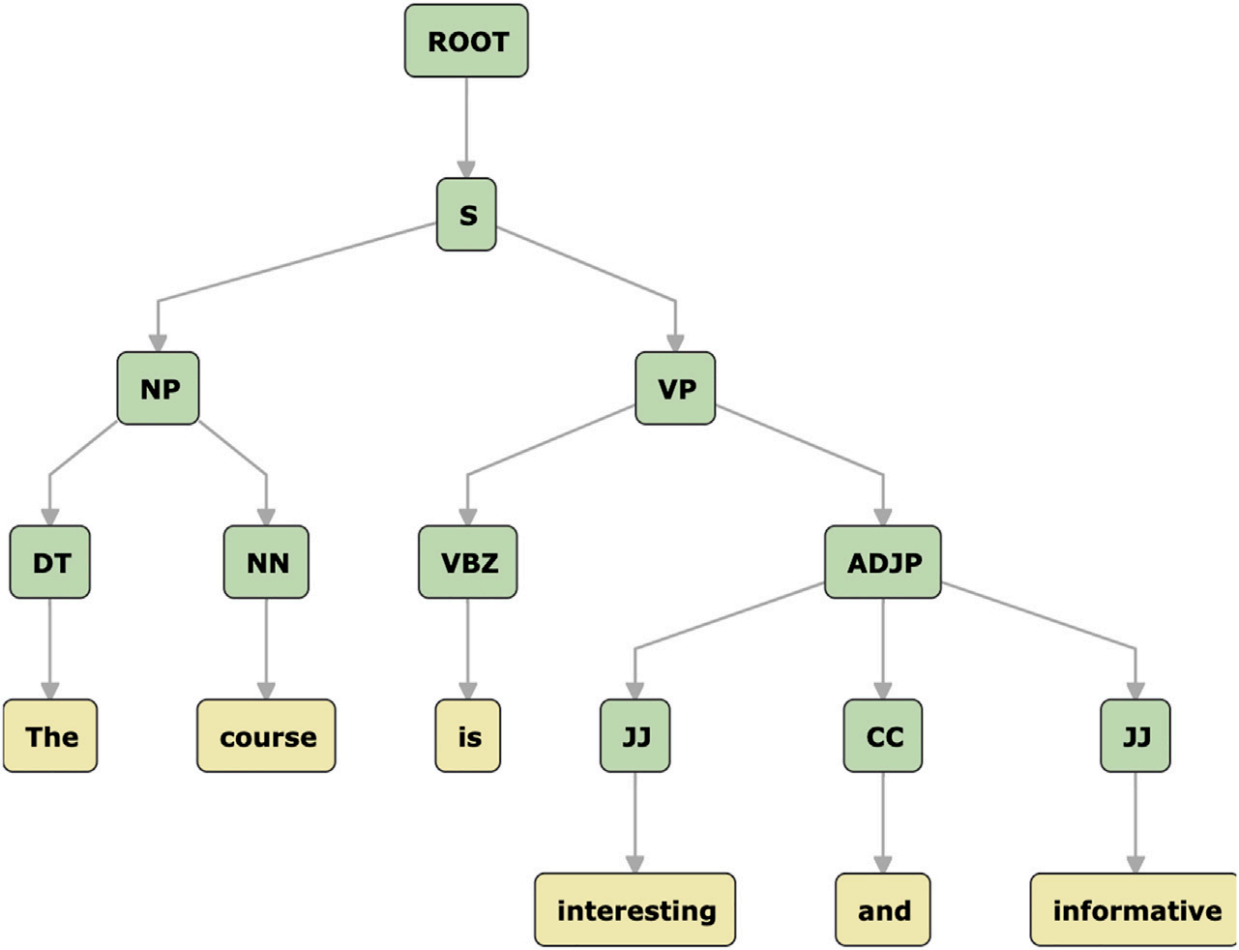
A constituency tree example based on PCFG parser.

As a step before training the neural networks, we created a pipeline of text normalization, to be used by every single model in our experiments to pre-process all comments. In other words, we expanded contraction, standardized URLs, punctuations, special characters, and corrected misspelled words. We have applied pre-processing steps that are commonly used for NLP tasks. More specifically, the pipeline steps were:

**Step 1**: firstly, as contractions often exists in the written form of English, we expanded these shortened versions of words in order to standardize the text (Mahmoudi et al., 2018). To illustrate, a phrase such as “I’ll be happy!”, becomes “I will be happy!”.
**Step 2**: we replaced all occurrences of URLs and hyperlinks by the string “URL” (Mahmoudi et al., 2018).
**Step 3**: special characters and punctuations can lead to noise in text; thus, we separated all non-alphanumeric characters from words (Conneau et al., 2016). For example, “Unfortunately,it’s a difficult course!” becomes “Unfortunately, it’s a difficult course !”.
**Step 4**: we used an adaptation of Peter Norvig’s spell checker^2^ to correct all typos on the comments.
**Step 5**: we applied a tokenizing technique onto comments (Sun et al., 2017). Resulting words/tokens then having numerical vectors representations with numeric indexes to our token sequences.
**Step 6**: we applied the zero-padding strategy based on (Cheng, 2020), which creates identical vectors lengths for all comments. Using the length of the longest sequence (70 tokens) we applied padding to all sequences, to ensure an uniform vector size for all vectors in our data.
**Step 7**: for classical models (Support vector Machine (SVM) and logistic regression (LR), we applied the TF-IDF Based on n-gram. This generates TF-IDF vectors for each gram. It is well known in the NLP domain that n-gram models boost accuracy of classification, as they take into account sequences of words. For deeper representation of texts, we also have examined n-gram TF-IDF based on characters (n = 2, 3, for both words).
**Step 8**: **Semantic Representation**: We used word representations (GloVe) For our words inputs embeddings. We used the 300D vectors of GloVe (Pennington et al., 2014) as recommended by (Kim et al., 2019). It generates a matrix of words based on co-occurrence statistics. We used the pre-trained Glove algorithm. This gives pre-trained weights for the inputs (transfer learning) instead of the start of random wights (learning from scratch). These initial inputs are fed to sequential NNs models to provide the semantic information for in word-level stage, and we updated the weights for these inputs with learning rate of 0.1 during the training.
**Step 9**: Because we are concerned about the phrase level in the second model, we applied sentence tokens for each comment before applying the next two steps.
**Step 10**: **Syntactic Representation**: For the gender dataset, we applied additional pre-processing steps which. We used a parser, based on an expert-designed grammar, to handle the sentences/phrase level of the text. We specifically used the Stanford Probabilistic Context Free Grammar (PCFG) parser (Klein and Manning, 2003), as it is more accurate and it provides constituents of text plus tags at phrase level (like NP, VP, ADJP, etc. ^3^), Figure 2. Constituency parser has been approved to be effective on many related studies (Kim et al., 2019).

## 5 Deep Learning for Learners Profiling

**FIGURE 3:**
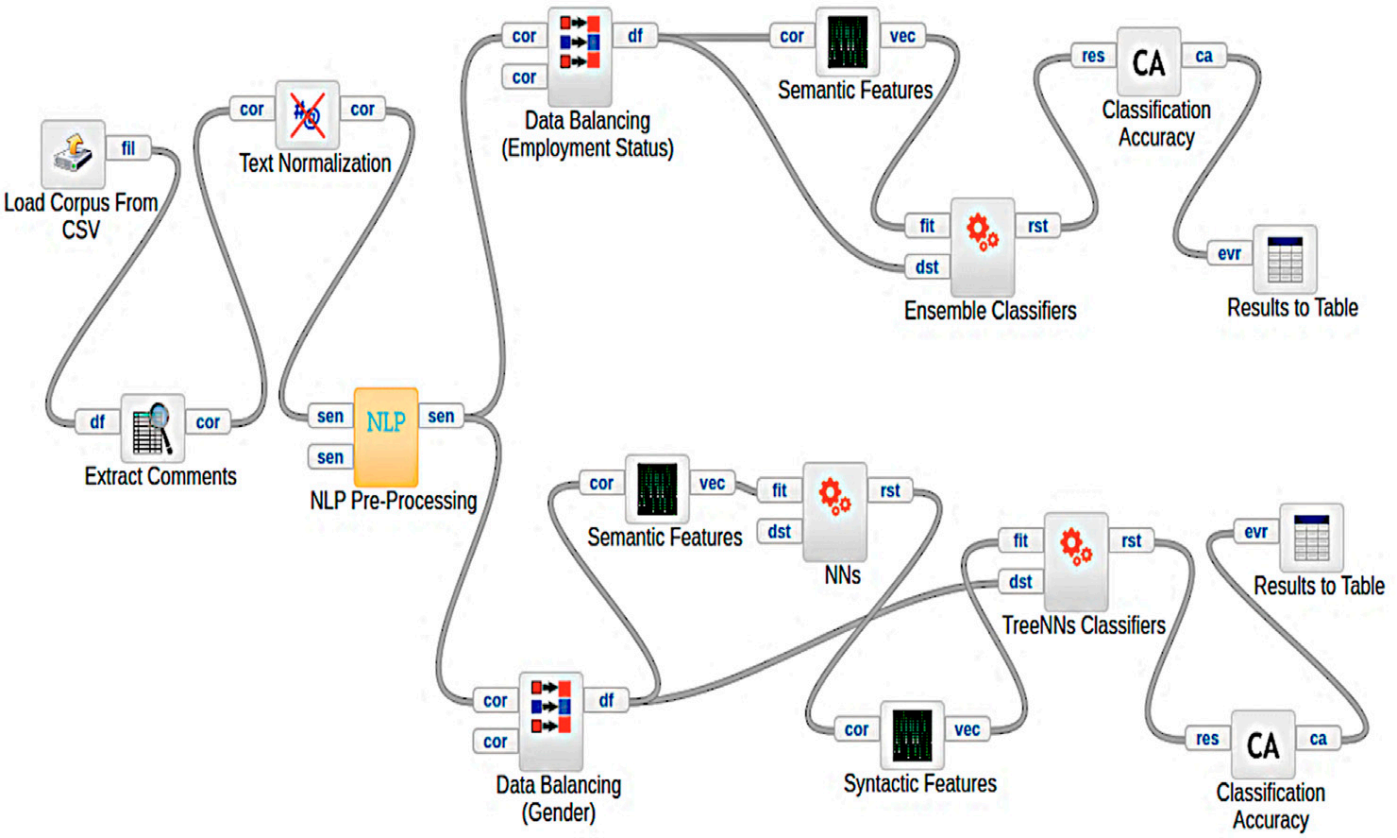
Our Approach general work flow (A frame diagram).

Even though classic machine learning that learned from probabilistic representations of samples of text has proven to be effective in AP tasks (Pardo and Rosso, 2019), deep learning has been a less examined solution for author profiling, according to our survey (Aljohani et al., 2020b). While AP is very important of task in NLP, there is less use of deep learning models for AP. For example, data provided in PAN 2019 (Pardo and Rosso, 2019) was very large data for AP tasks, however, only three studies out of 55 considering deep learning models, which is extremely low. This could indicate the difficulty of using NN model solutions for AP. Deep learning models are widespread solutions in NLP tasks nowadays and hence we considered them for our task. This is feasible in our study because we have enormous large enough samples from a specific domain (MOOCs). We also compare our models with baseline models (classical ML). For example, SVM is considered state of the art for occupation classification in previous studies, while logistic regression is the state of the art for gender classification. Figure 3 shows our general workflow for both models in our study.

## 6 Employment Status Profiling

**FIGURE 4:**
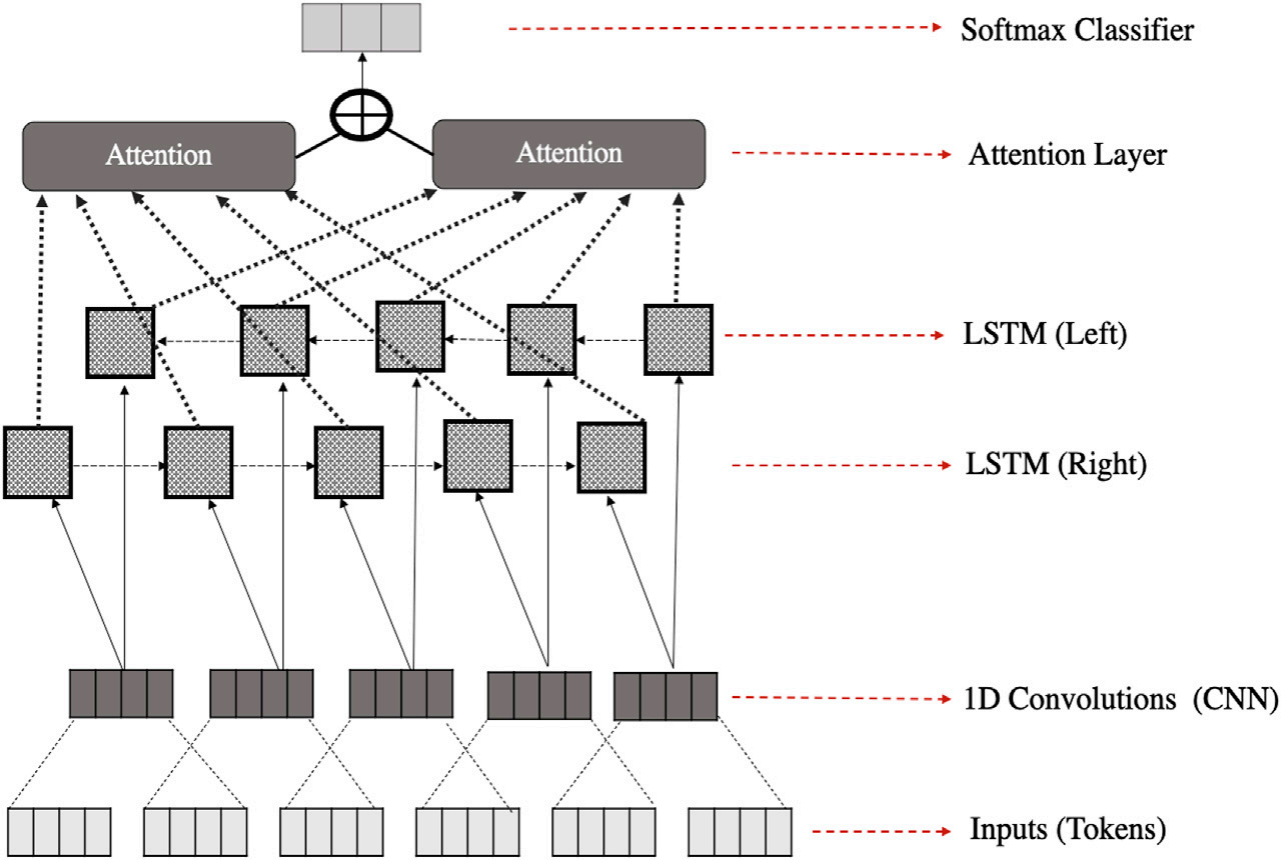
The sequential ensemble learning architecture.

To increase the chance of getting a high accuracy by using deep learning for the AP task, we selected to examine models that were previously proven to be effective in the NLP classification tasks (Yin et al., 2017a). Parallel Ensembling of CNN and LSTM have shown higher accuracies on other NLP task and on benchmark data: IMDB: (91.8) Subj (94.0), and TREC (97.0), Deng et al. (2021). An ensemble model combining CNN and RNN has been proposed relatively recently for author profiling. Our ensemble model is inspired by work done by (Cliche, 2017), who used both CNN and RNN by means of an ensemble deep learning architecture in a sequential manner. Their model is a text classifier model for the sentiment analysis task. However, we apply this to our AP task. A standard architecture of an ensemble learning CNN and RNN is represented in Figure 3. We have adjusted their original model to be used for our data and task. The model architecture is described in the following sections. In our study, we compare the performance of parallel ensembling and sequential ensembling. The sequential ensembling gives us better results, and its architecture is presented in Figure 4.

### 6.1 Embedding

The first layer in the model is the embedding layer, which maps each comment sequence onto a real vector domain. Thus, an entire comment representation (*X*) is mapped to a matrix of size $s\times d:X\in R^{s\times d}$; where *s* is the maximum number of words in the longest comment (*s* = 70), and *d* is the embedding space dimension. In addition, it is common in text classification to have 256-dimensional, 512-dimensional, or 1024-dimensional word embedding (batch size) (Aljohani et al., 2020a), when the data size is large. We found that *d* = 512 works well for our model, as it yield better results and accuracy comparing to 1,024 and 256-dimensional.

### 6.2 Convolutional Neural Networks

The second layer is a hidden layer, containing the convolutional model. A convolution ci applies a non-linear function *f* as follows:

$$ci=f\left({\text{Σ}}_{j,k}w_{j,k}{\left(x_{\left[i:i+h1\right]}\right)}_{j,k}+b\right)$$ (1)

Where *i* is the current input vector, *j* a position in the convolution kernel/filter *k*, and *h* is the number of words in spans (size of the convolution), *b* a bias term, *w* a weight and *x* is the current word embedding; (i:i) represents a sub-matrix of *x* (Cliche, 2017).

We have tried different settings in tuning parameters in the used model, but we will explain the final version of these settings. We have used 1D convolutional NNs, we have set the strides to be equal ones, and we applied valid padding. We have applied various filter sizes and filtering matrices. Our model has improved with filter size of (3, 4, 5), and with 200 filtering matrixes, as also recommended by (Cliche, 2017). For pooling, we used the widely applied max-pooling process. This extracts the most important n-grams within the embedding space. The max-pooling operation also provides a combination to all pooling in each filter into one vector. For instance, the total number of filters is: 3 × 200 = 600. The final vector obtained is fed to a fully connected layer (FC). We have experimented with different numbers of neurons, i.e., gradually from 10 to 50 neurons in the FC, followed by ReLU as an activation function. To reduce overfitting, we add a dropout hyperparameter, which is randomly removing words in sentences and forcing the classification to not rely on any individual words, to avoid overfitting; we set the dropout value to 0.5, which is considered a good traditional dropout setting for many NNs and tasks, according to (Srivastava et al., 2014). The final merged output matrix that is the output of the CNN model is fed as input to the RNN model, as part of the sequence in the ensemble learning. It is worth to mentioning here that a recent study (Sun et al., 2017) demonstrated that the CNN model is more effective in an embedding space represented by n-grams. This is due to the fact that it is not required in CNNs to have any knowledge of the syntactic structure of the language. Additionally, CNNs perform well in online data texts, as they are good in handling independent features, like new words in a language (Zhang et al., 2015). As we work with word tokens, NN were thus chosen.

### 6.3 Recurrent Neural Networks

The RNN model consists of a bidirectional Long Short-Term Memory (BiLSTM), as further explained, plus an attention mechanism. RNN models are considered effective for sequence modeling, such as analyzing a sequence of words. They handle sequential aspects of the data (Yin et al., 2017b), based on the position/time index in a sentence (Bhardwaj et al., 2018), and also tokens semantics. However, they are still not effective enough to handle small parts of texts, like n-grams, compared to CNNs. As a result, a combination of the CNN and RNN in an ensemble technique could provide complementary information about the author writing features, modeling semantic information of a text globally and locally (Chen et al., 2017). However, “vanilla” RNNs are known to have the vanishing gradient problem. Thus, LSTMs are chosen, as they can solve this problem due to their complex internal structure, able to remember either long-term or short-term information (Hochreiter and Schmidhuber, 1997). The following formulas briefly describe memories/gates inside a hidden unit of an LSTM that help the model to remember term information:

$$\begin{array}{l} f_{t}=\sigma \left(w_{f}x_{t}+u_{f}h_{t-1}+b_{f}\right)\\ i_{t}=\sigma \left(w_{i}x_{t}+u_{i}h_{t-1}+b_{i}\right)\\ o_{t}=\sigma \left(w_{o}x_{t}+u_{o}h_{t-1}+b_{o}\right)\\ c_{t}=f_{t}\circ c_{t-1}+i_{t}\circ tanh\left(w_{c}x_{t}+u_{c}h_{t-1}+b_{c}\right)\\ h_{t}=o_{t}\circ tanh\left(c_{t}\right) \end{array}$$ (2)

Where *t* is the timestep, *h* is a hidden state, $f_{t}$ is the “forget gate”, $i_{t}$ is the input gate, $c_{t}$ is the cell state, *u* is the weighted matrics. *b* is bias term, *w* is the weight term in these functions, σ is the sigmoid function, and *o* is Hadamard product (Zaremba et al., 2014) and (Cliche, 2017). To further enhance the LSTM structure and make it able to take past word information into account, we use the bidirectional strategy, which means deploying two LSTMs to feed our data inputs in two different directions (one to read sequences forward, the other to read sequences backwards, in other words, read from past to future and from future to past), plus the attention mechanism. Inputs of the two-LSTMs then will stack together, for better understanding of token sequences. The BiLSTM in our model has 100 hidden units in total (50 neurons in each direction), and a dropout layer with a rate of 0.5 to regularize learning; followed by a FC layer of 30 units and activation functions (ReLU). The FC layer is also followed by a dropout layer (0.5).

### 6.4 Classification Layer

Then, we use the flattening layer for the representation of the output data to be fed into a softmax classifier (final classification layer). The softmax function is best used with the last layer of prediction (Duan et al., 2003), as it uses the probability distribution of categories as a set of numbers between zero and one, whose sum is one. We compile the model using the Adam optimiser and a loss function (Kullback Leibler divergence; and categorical crossentropy produced similar results in our model) because we have three target categories in our task. In summary, the token representations, in our best performing model, are fed the CNNs to extract the most important embedded tokens. Next, the CNN layer outputs become the inputs for the Bi-LSTM, which is important for handling the sequencing of the data. This transfer is simply done by sharing the internal weights of neurons through the input sequence (Zaremba et al., 2014) and (Cliche, 2017). Followed by the mechanism technique, to get the most important final information representation. After this, we used a simple classification output layer (Softmax) to predict learners’ employment status, Figure 4.

## 6.5 Results and Discussion

To assess the ensemble models in a comprehensive and realistic way, we used different performance measurements: accuracy, F1-score, precision recall, and confusion matrices as well. We use these evaluation metrics as they represent the most popular measurements of performance for classification tasks. Utilizing them all provides a full picture of the range of performance for each model. We applied classical machine learning (SVM). Also, we compare our model performance with a CNN model, as well as an RNN model, to confirm our intuition that an ensemble method is more appropriate for this task than a “simple” deep model. In addition, we compared our sequential ensemble learning model with the traditional parallel ensemble deep learning architecture. For fairness in comparisons, all deep learning models applied in our experiments use the exact same parameters as the above described ones. Furthermore, we compare the performance of these four models based on the two balancing methods that have been used. i.e., for each model, we applied Random Oversampling (R.O), as well as the paraphrasing technique, Text Augmentation (T.A). We summarize the highest accuracy results of two ensemble models in our experiments in Table ? We obtained these highest results when we have applied the paraphrasing technique for balancing the data. We present the results of each model involved in our experiments in Table 1 for the validation dataset. The validation data set is extracted separately from the training data, based on comments from a different group of learners, to avoid bias of during learning. Therefore, 10-fold cross-validation is not needed (Kim et al., 2019), and such method recommended when training samples are small or validation data is from training data, However, our data size is huge in size.

**TABLE 1:** All models with overall accuracy results.

Model	Accuracy (%)
SVM (n-gram TF-IDF) with random oversampling	64.3
SVM (n-gram TF-IDF) with text augmentation	71.8
CNN model with random oversampling	93.4
CNN model with text augmentation	92.2
RNN model with random oversampling	76.3
RNN model with text augmentation	78.5
Enasemble parallel model with random oversampling	87.2
Enasemble parallel model with text augmentation	90.3
Enasemble sequential model with random oversampling	81.3
Enasemble sequential model with text augmentation	**96.4**

The results are represented by the ratio of the correctly predicted samples to the total samples in our data (Accuracy). In second Table, we furthure provide results details of much higher accuracy Model, as our data originally was unbalance. The first column represents the Precision, which is the ratio of the number of true positives (TP) divided by the sum of the number of false positives (TP) and the number of false positives (FP). Recall in the next column represents the ratio of the (TP) divided by the sum of the (TP) and FN (false negative). Followed by F1-score, which is a weighted mean of both precision and recall. These results are represented by the ratio of the correctly predicted samples to the total samples in our data, which is called Accuracy. The equations below explain these evaluation methods mathematically:

$$Precision=\frac{TP}{TP\;+FP}$$ (3)

$$Recall=\frac{TP}{TP\;+FN}$$ (4)

$$F1=\frac{2TP}{2TP\;+FP+FN}$$ (5)

$$Accuracy=\frac{TP+TN}{TP\;+FP+FN+TN}$$ (6)

The implementation of all the models is done *via* Python libraries for NNs. We perform 40 iterations of training for each model in our experiments. Table 1 shows that we have obtained 96.4% overall accuracy on the validation dataset that is collected, as explained in data Section 3.2, that predicted the employment status of learners. The CNN performance is superior to that of the RNN, even when they were training on an identical embedding layer. Surprisingly, we also found that CNN performs better than the parallel ensemble learning. Further experiments are required to investigate this. On the other hand, sequential ensemble learning has achieved better results than parallel ensemble learning. The other important point to make is that all models, without exception, have achieved higher results when we applied the paraphrasing strategy. In general, the power of our data size during training can be recognized, because of the high results of almost all deep learning models that have been used in this study. The worst is RNN, still with high accuracy of 76.3%, as can be seen in Table 1. In Table 2, we provide further details about the parallel model’s performance for each category of the employment status, while Table 1 shows the same information, for the sequential model. It is important in this section to not report only average results, which can thus be strongly biased, but also detailed and non-equivocal results for our class categories (target: employment status) and the model results for each category. As can be seen in Table 2, even at the detailed category level, our model performs exceptionally well. We can conclude from our experimental results that using sequential architecture in ensemble models for learning data representations in our task, associated with the paraphrasing strategy to balance data, can perform well for such a prediction.

**TABLE 2:** Parallel and Sequential model with TA: Results on each class target (in %).

Employment status	Parallel model			Sequential model		
	Precision	Recall	F1-score	Precision	Recall	F1-score
Working	90	91	90	98	95	97
Not working	83	92	87	98	97	97
Retired	93	89	91	93	97	95

Overall, we have designed our model with awareness of computational issues. Whilst our model, as an ensemble model, can introduce a level of complexity, we have strived to reduce this by only considering simple inputs (e.g.: tokens, instead of complex stylometric features) to represent the data, which can reduce computation time and storage. In our solution, we combine the benefits of the NLP and DL models to predict the learners’ current job situation only by using their comments. The further, next step goal is providing recommendations that support learners and meet their needs. We could develop an adaptive interface based on the learner’s work/professional needs, based on the ample literature in this area. For our case, this translates into course material and other recommendations based on learners’ employment status, which could increase the engagement among learners in MOOCs. For instance, providing retired learners with additional reading, under the assumption that they may have the time available to do so; or providing “not working” users information about what type of jobs are related to the current course, etc. (Aljohani and Cristea, 2019).

## 7 Gender Profiling

### 7.1 Recursive Neural Networks (RvNNs)

It has been indicated in the literature review that gender is more difficult to predict compared with other users’ characteristics, even if it is performed as a binary classification task (Aljohani et al., 2020b). User Profiling based on gender is the most investigated task among other tasks in the AP literature review. Extensive examinations of both traditional and deep learning models have been done to predict users’ gender from their texts. To the best of our knowledge, there is no AP study has consider the recursive approach of text to predict gender of users. It has been only applied for users relationships on twitter (Mac Kim et al., 2017), not based on textual features. Gender prediction has been studies before based on RNN and CNN. So we have studied another text representation and approach, which is a recursive representation, to handle the gender profile. While syntactic forms of textual data representations has been studied for gender profiling for example based on part speech tagging, the syntactic representation based on tree structure of text has not been widely explored in AP. It is mainly a phrase level processing of a sentence that is structured as a tree, based on its constituents (Figure 2), and this is different from a words/sequences-based sentence structure. Few works have been done in NLP that used the syntactic representation based on tree-structured deep learning to explore treasured information that are associated with syntactic parse, as they are represent a sentence meaning as well. So, sentence encoded commonly as sequence-based tokens in recurrent neural network that based on LSTM (Hochreiter and Schmidhuber, 1997) in which information is accumulated sequentially over a sentence; convolutional-based neural networks (Kalchbrenner et al., 2014) in which information is accumulated using filters to perform this over short local tokens/sequences of either words or characters; or lastly a tree-based structure in recursive neural networks Goller and Kuchler (1996) and (Socher et al., 2011) in which information is propagated up binary tree parse. Among these three styles of models, Tree structure models is a principle option as language meaning naturally constructed in tree/recursive form (Dowty, 2007). This representation of text provides a comprehensive interpretation for a sentence meaning. According to a linguistic principle, a sentence in natural language can be presented as a set of components that are nested constituently in a tree structure form (Partee et al., 2012). Thus, can be extracted by a parser model trained on identified treebanks, providing a constituency trees that are handy in many sentence level related tasks. Covert a plain sentence to a semantic representation is a fundamental process in many NLP, and deep Learning Models is powerful in produce such representation of information either at word-level or sentence-level. The basic approach with many NLP models NLP that is to represent the text as a sequence of words (flat way). However, languages has different information structure and they could also be consider in AP. The natural structure for language is the tree structure; which is based on usually based on a language grammar.

Recursive neural networks (RvNNs) models have be designed to handle such a textual structure and reflect its syntactic representation. These designs have been achieving magnificent results on numbers of sentence classification problems, like natural language inference (Bowman et al., 2015), sentiment analysis (Socher et al., 2012), and discourse relation classification (Wang et al., 2018). RecNNs are used in NLP to represent a sentence as a tree/recursive structure. This structure can be learned during training or given by a parse tree; and the latter is commonly used as it has been shown to be effective Kim et al. (2019). A RecNN model converts an input word to a vector, which is a leaf node; and nodes pairs then composes into phrase pairs by a composition function. This called an intermediate representation of a tree. Lastly, the root node is considered the representation of the whole sentence.

Although pure TreeRNN, which only reach the closest constituent parts within a sentence, is more effective in term of getting the meaning composition of a sentence, however, it still reaches a limited space of information in a sentence and did not got the whole the big picture of the sentence meaning. Tree-sequential is useful to process human-wize during reading a sentence. from left to right and this can provide the whole picture based on current steps in the tracking vector of LSTM. However, adding a transition process during encoding a sentence in dynamical way. This help to improve the sentence understating more. During the sequential process of words sequences, the model will have current status of a word that summarizes the whole left context, and by this summarizes some of information has lost which bring some disambiguation before reaching the last word of the sentence. However, tree-structured models start with constituent of a sentence that has its merged words (Bowman et al., 2015). To produce better tree-structured models, previous NLP researches have examined sequential models that used to achieve state-of-the-art results, and they extend sequential models then comparing their performance (Tai et al., 2015). LSTM, the most powerful NN architecture in NLP, due to its superiority in memorize long length sequences, It has proven also to be effective in its new expanded version (TreeLSTM).

These models have been explored only marginally for text classification, and have not yet been applied at all for author profiling, to the best of our knowledge. Previous studies in AP have considered a syntactic representation of text such as POS, but it simply examined either in order techniques such as bag-of-words models or in a sequential technique such as recurrent NN models. These models, however, are not fully sufficient to capture the text semantics because they do not taking into account the ambiguous in of natural language. For example, a sentence like I saw the person with the telescope, can have two meanings: I saw the person (with the telescope), which mean I saw the peson who had a telescope, or I (saw the person) (with the telescope) I used the telescope to view the person, which means I used the telescope to see the person. This because it is normally in languages that sentence can have different types of structures, which bring differences in meaning. Such differences can be capture with syntactic representations. Tree-structured models are an optimal choice to interpret such text representation of a sentence. We have recognized the bleeding edge models, which is TreeLSTM based models, and applied them, for the first time, for an AP task.

### 7.2 Tree Based Neural Networks

Earlier recursive neural network models, such as Tree-LSTM is the first model of these kind to be presented to pass tree structured information over sequences (Tai et al., 2015), but they well-known for having a long training time and having difficulties in utilizing the advantages of batch-computation, due to the diverse complex structure of sentences.

In 2016, the Stack-augmented Parser-Interpreter Neural Network (SPINN) model was introduced (Bowman et al., 2015), to allow efficient recursive neural network training, by adopting the idea of a shift-reduce parser from the compiler (Aho and Ullman, 1972); this is still the state-of-art model since 2015. In this same paper, a different composition function to construct the tree was introduced and it increased the training accuracy and testing accuracy by 5.3 and 2.6%, respectively, on the Natural Language Inference dataset (NLI), compared to the baseline model LSTM RNN. This new composition function has an extra input for information, which is generated in real-time during the encoding of the sentence. SPINN is of the most promising and highly cited method for structured language processing on syntactic supervision learning SPINN increased the speed of learning for tree-structured models, allowing thus handling large-scale NLP tasks, since previous models couldn’t support batched computation. Also, the major problem for recursive neural networks still remains that the network only reached local optimization at each node instead of reaching a global optimum at the root of the tree. SPINN introduced a solution called tracker, which aims to summarize the sentence information during training. This information provides higher accuracy, but it can only summarize limited information in a sentence.

In 2019, the SATA (Structure-Aware Tag Augmented)-Tree-LSTM model was proposed, addressing this limitation, it introduced additional information and used a separate LSTM tree to model the sentence, which empirically reached a better optimum over the tree (Kim et al., 2019). SATA is also a state-of-the-art model in the area. However, in the SATA model, the extra information only contributes to the gate information. The SATA Tree LSTM model has achieved state-of-the-art accuracy results in four out of five public datasets (Kim et al., 2019).

We have designed an ablation study by comparing several versions of these tree-structured LSTM models, as well as providing our novel version of bi-directional composition function for existing architectures (SPINN/SATA). In total, we have evaluated 18 different architectures of phrase-level encoding function on our dataset to predict the gender class of learners.

#### 7.2.1 TreeLSTM

**FIGURE 5:**
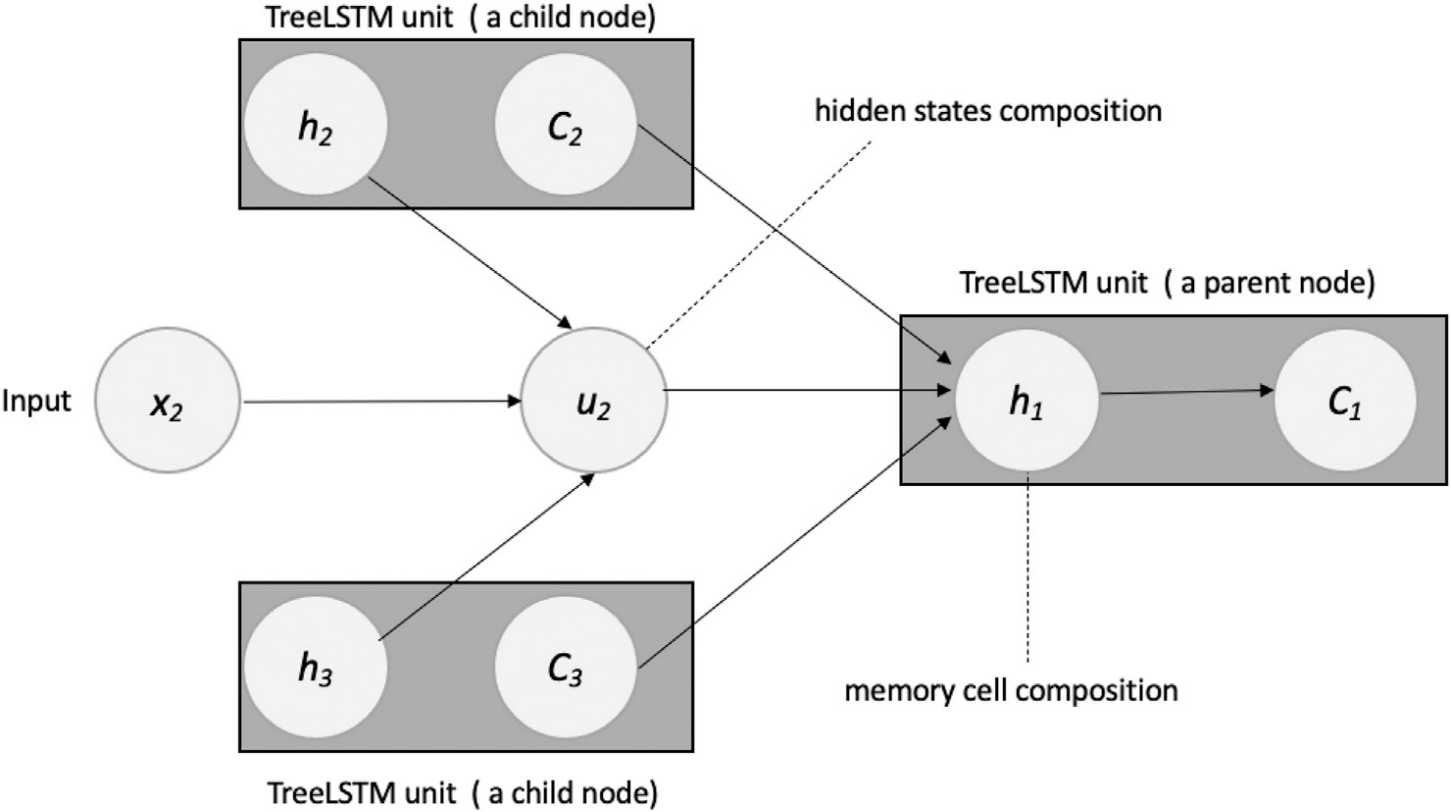
Composing: memory cell and hidden state of a Tree-LSTM unit: Two children nodes (2 and 3).

The hidden state of the original LSTM is composed of a current input at a current time step and a previous hidden state of an LSTM unit in the previous time step. However, the hidden state of the tree LSTM is composed of a current input vector and hidden states of two child units (in the case of a binary tree) (See Figure 5).

In a standard tree-structured LSTM cell, the composition functions are as follows:

$$\left[\begin{array}{c} \begin{array}{c} \begin{array}{c} \begin{array}{c} i\\ f_{l} \end{array}\\ f_{r}\\ o \end{array}\\ g \end{array} \end{array}\right]=\left[\begin{array}{c} \begin{array}{c} \begin{array}{c} \begin{array}{c} \sigma \\ \sigma \end{array}\\ \sigma \\ \sigma \end{array}\\ tanh \end{array} \end{array}\right]\;\;\left(w\left[\begin{array}{c} h_{1}\\ h_{r} \end{array}\right]+b\right)$$ (7)

$$C=f_{l}\;⊙\;c_{l}+f_{r}\;⊙c_{r}+i\;⊙g$$ (8)

h=o⊙tanh(c) (9)

Where h,c∈Rd refers to the hidden state and cell state, respectively, in the current cell; in tree-structured LSTM, $h_{l},h_{r},c_{l},c_{r}\in Rd$ represent the hidden states and cell states of a pair of child nodes (left and right); g∈Rd refers to the composed inputs from both children and $i,f_{l},f_{r},o\in Rd$ represent input gate, two forget gates and an output gate, respectively. These two separate forget gates from two children allow the network to choose to forget different information in each child, which captures a more complex representation of the information from the same sentence. w∈R5d×2d and b∈R5d are trainable parameters in the model, σ and tanh refer to sigmoid and hyperbolic tangent functions, which apply non-linear transformations before the gate information is updated, and ⊙ is the element-wize multiplication symbol, as the dimensionality of elements on both sides is the same. The equations here refer to a binary tree; however, tree-structured LSTM is not limited to two-children cases and can be easily extended to multiple children cases, due to the flexible nature of the recursive neural network. In this research, we adopted a binary tree setting, which is mostly used in related literature.

### 7.3 The Stack-Augmented Parser-Interpreter Neural Network Model

**FIGURE 6:**
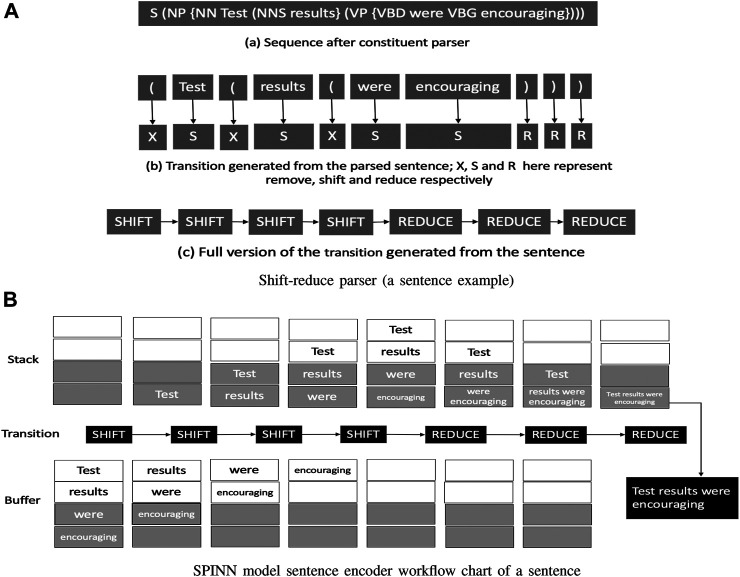
SPINN model architecture.

This model combines both parsing and the interpretation concept into hybrid model, which integrates the interpretation of the tree-structured texts (sentences) with a linear sequential parser (shift-reduce parser) within a single tree sequence model (The hybrid). They proposed to have a hybrid model because in human language it is common to have multiple meanings for the same word, depending on the text content, which is called Lexical ambiguity. The SPINN model provides a way to reconstruct the complex syntactic structure of the language by reading it from left to right with the help of a shift-reduce parsing algorithm (Aho and Ullman, 1972). The shift-reduce algorithm takes a sequence of inputs with length N and converts it to 2N−1 length transitions, as shown in Figure 6A; the sequence of transitions is either shift or reduce. Then, the sequences of words from the sentence and related transitions are fed into the SPINN model. To encode the complex structure of the tree, two data structures are used, which are called stack and buffer, both with size N. In the beginning, the sequence of inputs is fed into the buffer in order; when the transition is SHIFT, the top word in the buffer is pushed to the bottom of the stack and when the transition is REDUCE, the bottom two words in the stack are popped out and combined into one word; then this new word is pushed to the bottom of the stack, as shown in Figure 6B.

The composition function used in SPINN is different compared to the traditional Tree LSTM function, as it introduced a component called tracking LSTM which is denoted as *e*. It increased the training accuracy and testing accuracy by 5.3 and 2.6%, respectively, on the Natural Language Inference dataset (NLI), compared to the baseline LSTM RNN. This piece of extra input information is generated in real-time through the sentence-encoding process, Figure 6B; it consists of three components: two word-level-embedding from the two bottom positions of the stack and one-word-level embedding from the top position of the buffer. This extra information *e* provides a representation of the current status of the sentence encoding process, and also the current status of the buffer and stack. In addition, it supplies more information to the composition function. To generate e from the three components from the stack and buffer, a simple linear mapping is used. This information provides a global datum in each current cell, so it can expend the information in each step. It works as an indicator for the progress of the sentence encoding. Thus, SPINN represents another excellent candidate for our author profiling for the gender of learners in MOOCs. The composition function for SPINN is shown as below:

$$\left[\begin{array}{c} \begin{array}{c} \begin{array}{c} \begin{array}{c} i\\ f_{l} \end{array}\\ f_{r}\\ o \end{array}\\ g \end{array} \end{array}\right]=\left[\begin{array}{c} \begin{array}{c} \begin{array}{c} \begin{array}{c} \sigma \\ \sigma \end{array}\\ \sigma \\ \sigma \end{array}\\ tanh \end{array} \end{array}\right]\;\;\left(w\left[\begin{array}{c} h_{1}\\ h_{r}\\ e \end{array}\right]+b\right)$$ (10)

$$C=f_{l}\;⊙\;c_{l}+f_{r}\;⊙c_{r}+i\;⊙g$$ (11)

h=o⊙tanh(c) (12)

### 7.4 The SATA Model

The SATA model is motivated by the tree LSTM and SPINN models, but it provides different extra information to the model from a tag representation, which is generated as a by-product by the parser and creates an extra LSTM network to learn a higher representation of the tag at each node. This information from the new LSTM model is equivalent to the tracker LSTM part, which is originally mentioned in the SPINN model. This new piece of input information that is taken from tags shares the same idea of a tracker LSTM, which is a representation of the current state for the encoding process (which is the level of the tree structure) and adds more information to the tree-LSTM encoding function. In addition, this provides more information on the syntactic structure of the sentence; however, this time, the extra information only contributes to the gate-information in the LSTM cell and does not influence the actual input information in the composition function, as shown below:

$$\left[\begin{array}{c} \begin{array}{c} \begin{array}{c} \begin{array}{c} i_{1}\\ f_{l1} \end{array}\\ f_{r1}\\ o_{1} \end{array}\\ g \end{array} \end{array}\right]=\left[\begin{array}{c} \begin{array}{c} \begin{array}{c} \begin{array}{c} \sigma \\ \sigma \end{array}\\ \sigma \\ \sigma \end{array}\\ tanh \end{array} \end{array}\right]\;\;\left(w\left[\begin{array}{c} h_{l}\\ h_{r} \end{array}\right]+b\right)$$ (13)

$$\left[\begin{array}{c} \begin{array}{c} \begin{array}{c} \begin{array}{c} i_{2}\\ f_{l2} \end{array}\\ f_{r2}\\ o_{2} \end{array} \end{array} \end{array}\right]=\left[\begin{array}{c} \begin{array}{c} \begin{array}{c} \begin{array}{c} \sigma \\ \sigma \end{array}\\ \sigma \\ \sigma \end{array}\\ tanh \end{array} \end{array}\right]\;\;\left(w\left[\begin{array}{c} e \end{array}\right]+b\right)$$ (14)

$$c=\left(f_{l1}+f_{2l}\right)\;⊙\;c_{1}+\left(f_{r1}+f_{r2}\right)\;⊙c_{r}+\left(i_{1}+\;i_{2}\right)\;⊙g$$ (15)

$$h=o_{1}+o_{2}⊙tanh\left(c\right)$$ (16)

The SATA model proves that by using more linguistic information, such as tag information, it helps in sentence understanding. Also, one of the advantages is that the SATA model allows for dynamic composition of the language tree, which can use all information of each single sentence without losing any information. The original SATA tree used a difference encoding function for the leaf node and non-leaf node as it used extra information, which is the tag, compared to other existing literature. They test thier model on five public datasets and perform a state of the art on all of them. Which is support a claim that linguistic priors are advantageous to use for sentence interpretation tasks. In addition, it is known that chance of having higher accuracy in deep learning models increases when more correct encoded information is fed to the model. In deep learning research in general, the more input information is fed to the model, the better performance the model can show, as it reduces the uncertainty of the model, by providing extra information. Thus, SATA represents the most current, bleeding-edge state of the art, and hence is our final candidate for gender-profiling in MOOCs.

## 7.5 Results and Discussion

We tackle here the difficult problem of predicting the gender of learners based on their comments only which are often available across MOOCs. It is one of the most common metadata used for analyzing MOOC platforms, due to its wide application and use, is the discussion forum (Aljohani et al., 2020b). Forums are used for learning and social interactions, providing rich metadata to study learners and their needs. The three models provide different versions of composition functions with extra input, which indicates the state of the encoding process over each sentence. The results confirm the advantages of using this extra information to construct the tree-structured model; however, there is a gap in comparing the effect of using these different composition functions in sentence representation, especially for the classification problem in NLP. Thus, we have compared eighteen different combinations of composition functions of sentence encoding methods in our classification problem (gender classification).

We have evaluated the performance of these models, along with an LSTM model baseline, which is usually considered as a deep learning baseline in such experiments (Merrill et al., 2019). Parameters of all deep models in our experiments as the same, they are as follows: 25 minibatch, 0.05 learning rate, per-minibatch L2 regularization, and 0.5 dropout, AdaGrad optimizer, and softmax classifer. In addition, we found that the performance of the model is better when we split comments into sentences, and pad each sentence length to be of 20 tokens, maximum.

We considered the Tree LSTM model, which does not include tags during learning and this valid because our data is large; as some studies indicated that large data size could help neural models to learn syntactic rules, even without including tags during learning, which means that no need for external morphological information when data size in large enough (Kim et al., 2019). Even though we considered the tags *via* the other two models as we aim to more expanded compassion.

Structured Tree LSTM architecture in general has two steps: word-level encoding with a feedforward neural network, or LSTM neural network; and sentence-level encoding with a tree-structured LSTM composition function. We applied the binary tress parser for our study as it used by the three models. We aimed to evaluate different effects of combining the word-level encoding function and sentence-level composition function given the same information, so only a partial idea is taken from SATA. In addition, whilst previous literature recommended using bi-directional LSTM for word-level encoding, there is no such work to introduce bi-directional LSTM for sentence-level encoding. Our work contributes thus to fill this gap, by introducing bi-directional LSTM in the sentence-level composition function and analyzing its results.

**FIGURE 7:**
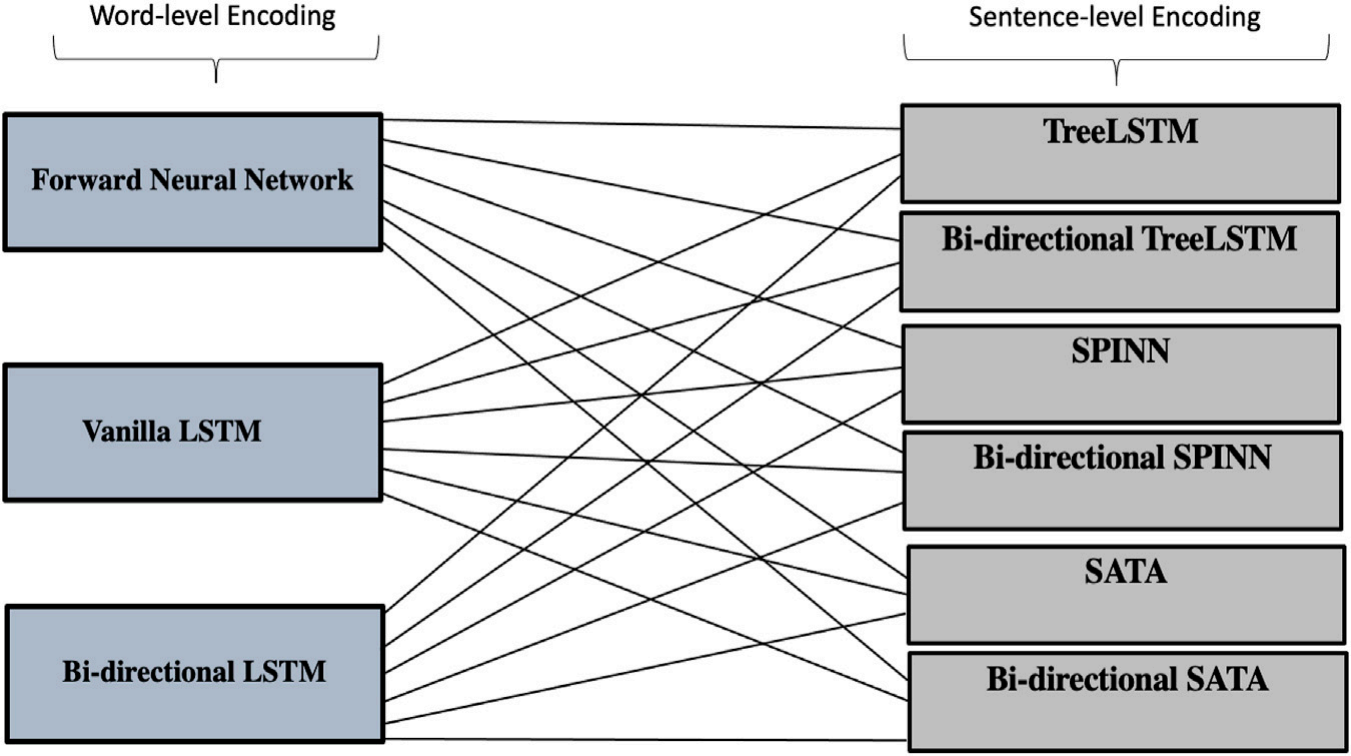
Our 18 combinations models, and our versions of Bi-directional SPINN and STATA.

The first step is word-level encoding and the encoding methods we evaluated are one hidden layer feed-forward neural network, basic vanilla LSTM neural network with one hidden layer and basic bi-directional LSTM neural network with one hidden layer. The second step is sentence-level encoding, which constructs the structured LSTM tree with a different composition function. Four different versions of the composition function are evaluated in this research which are: the LSTM tree, composition function taken from the SPINN model, from the SATA model; and their bi-directional composition functions (our novel version). Figure 7.

It has been found that updating Glove vectors for word-level encoding gives minor gain on binary classification tasks with the Treelstm models (Kim et al., 2019). However, we have a huge data size, and updating the initialized Glove vectors during training yields a boost performance in capture the more accurate semantic of the contact. This due to the fact some words meaning in the educational domain is different compared with others domains; such as a program, a course, a degree, etc. Updated these word vectors, although it creates different embedding dimensional which may higher, yet it boosts the classification performance. We implemented all the models we mentioned above in the same settings. We also compare our models against the standers LR, as classical ML, and LSTM baseline, as a recurrent mood, and our recuresive mood model is higher that the recurrent mood by 5%. We evaluated the results of the models on our data and report the test accuracy results in Table 3.

**TABLE 3:** Accuracy results of the syntactic models (in %).

Model	Classification accuracy (%)
Logistic regression (n-gram TF-IDF)	75.0
Vanilla LSTM (traditional sequence)	78.80
Forward neural network + TreeLSTM+ With Bi-Directional composition functions	81.49
	81.67
Forward neural network neural network + SATA+ With Bi-Directional composition functions	81.90
	82.20
Forward neural network + SPINN+ With Bi-Directional composition functions	81.60
	**82.60**
Vanilla LSTM + TreeLSTM+ With Bi-Directional composition functions	81.60
	80.70
Vanilla LSTM + SATA+ With Bi-Directional composition functions	80.60
	81.49
Vanilla LSTM + SPINN+ With Bi-Directional composition functions	80.60
	80.99
Bi-directional LSTM + TreeLSTM+ With Bi-Directional composition functions	81.86
	82.49
Bi-directional LSTM + SATA+ With Bi-Directional composition functions	82.10
	82.17
Bi-directional LSTM + SPINN+ With Bi-Directional composition functions	81.47
	82.55

The results presented in table 3 is based on data validation accuracy. The validation data set is extracted separately from the training data, based on comments from different groups of learners, to avoid bias during learning. Therefore, 10-fold cross-validation or another evaluation technique is not needed (Kim et al., 2019), as such methods are recommended only when training samples are small or validation data is part of the training data-set. In addition, these models are very complex and they are taking days of training.

Based on the experimental results, our model achieves a competitive performance. In general, all models have achieved high performance in predicting the gender class (80% or above), expect baseline models (LR and LSTM). Results of the two versions of each model are similar, but our bi-directional composition function models have achieved slightly better results. This has confirmed that using phrase-level representation is very effective for learners gender prediction, which sheds further light on sentence classification tasks in the educational domain. The highest recorded result in our experiment is of 80.62%, which is achieved by our proposed new model based on the simple Forward Neural Network combined with the SPINN model. This indicates the importance of the extra information that the model obtains during the training, which is not necessary to be limited to tags of constituents. Also, we can see that using only a simple model with for word encoding is still able to achieve high results.

When comparing the models using different word-level encoding functions, the linear mapping (Glove) works best over other LSTM encoding methods. This might be due to the fact that using the linear mapping better preserves word-level semantics, while the LSTM encoding alters the semantic meaning at word-level, making it harder to structure the sentence from a syntactic perspective. This might also relate to the complexity of the task.

In addition, the results indicates the importance of the extra information that the model obtains during the training, which is not necessary to be limited to tags of constituents. Also, we can see that using only a simple model with for word encoding is still able to achieve excellent results. As the tracker LSTM in SPINN provides less information compared to SATA, this information may not contribute much when the task is complex, like in author profiling. However, by including more linguistic information, the accuracy did not really affected.

## 8 Strength and Weakness

We confidently can claim that our results are robust and reliable, thankful to our enormous data size. However, these models are complex and need satisfactory computational resources. In spite of that, deep learning models are emerging state of art in NLP and they don’t demand the heavily feature engineered that are required for the traditional machine learning, and in AP researches, using those traditional models principally done by experimenting with thousands of textual features to analysis authors writing styles.

In addition, the various steps we applied, such as augmentation, paraphrasing, could, in principle, allow for information loss. We have however experimented with leaving these steps out, and performance suffered as a consequence, suggesting that they were necessary. What is more, these models are complex and need sufficient computational resources. For example, Tree LSTM models, additionally to our Bi-directional model, increased this complexity even further. It yields competitive results, but training a Tree LSTM model with a huge dataset is time-consuming, where training could take to 6–7 days on a 16 GB memory GPU. Importantly, we apply author profiling to the critical domain of education, using MOOC data collected through the FutureLearn platform, and offering solution competitive to all cutting-edge models, based on a solid, comprehensive analysis as well as on a very large dataset. Thus, various stakeholders of computer-based education, such as administrators, implementers, researchers, practitioners, educators, teachers, and ultimately, students, could benefit from personalized learning environments tailored to their needs. The high accuracy, especially of the prediction over MOOC data, is particularly promising.

We also considered the ethical aspect in our research as our data is labeled *via* a self-report survey, collected by learners themselves, and we had their permission (*via* standard FutureLearn practice) of using this information for research purpose.

## 9 Conclusion

Comparing with other models deployed for predicting users’ demographic characteristics, we emphasize utilizing different textual features for solving the problem. Our models in this research processed tokens as textual features, and we also discovered the syntactic features of text. Nevertheless, our models were able to achieve comparable results only based on basic NLP normalization tools and using an innovative text-argumentation strategy, which has not been used before for the AP, followed by a sequence ensemble deep learning architecture. In addition, our novel version of the bi-directional strategy that we have applied for all the 18 models has achieved a higher result than every corresponding model. Directions for future work include investigating different features (non-textual features); as well as extending to various authors’ characteristics.

## Data Availability

The raw data supporting the conclusion of this article will be made available by the authors, without undue reservation.

## Footnotes

1
https://www.futurelearn.com/

2
https://pypi.org/project/pyspellchecker/

3
http://www.surdeanu.info/mihai/teaching/ista555-fall13/readings/PennTreebankConstituents.html
